# Cellulose and Its Nano-Derivatives as a Water-Repellent and Fire-Resistant Surface: A Review

**DOI:** 10.3390/ma15010082

**Published:** 2021-12-23

**Authors:** Mehrnoosh Tavakoli, Ali Ghasemian, Mohammad Reza Dehghani-Firouzabadi, Bartłomiej Mazela

**Affiliations:** 1 Department of Pulp and Paper Technology, Gorgan University of Agricultural Sciences & Natural Resources, Gorgan 4913815739, Iran or mehrnoosh.tavakoli@up.poznan.pl (M.T.); ghasemian@gau.ac.ir (A.G.); m_r_dehghani@mail.ru (M.R.D.-F.); 2 Faculty of Forestry and Wood Technology, Poznan University of Life Sciences, Wojska Polskiego 28, 60-637 Poznan, Poland

**Keywords:** cellulosic-based substances, nanocellulose, coating, water repellency, flame retardancy

## Abstract

The inevitable destructive effects of moisture and temperature are obvious in cellulosic and nanocellulosic substrates. These materials are the main foundations of interdependent industries that produce products such as currency notes or high-quality packaging for sanitary, cosmetics, or ammunition in the defense industry. Therefore, it is essential to develop procedures to eliminate problems arising from humidity and fire to improve the quality of these green and sustainable materials. The production of waterproof and flame-resistant cellulose-based substrates has drawn increasing attention to resolve these drawbacks. In this review paper, we have initially summarized the most accessible cellulosic substrates, different kinds of nanocellulose, and the general information about water repellents and intumescent fireproof surfaces. Then, the potential and necessity of using cellulosic biobased substrates are addressed for use in modified shapes as waterproof and fire inhibitor coatings. Cost-effective, eco-friendly, and durable, dual-function coatings are also introduced as future challenges, which are exploited as water-repellents and flame-retardant cellulose-based surfaces for pulp and paper applications.

## 1. Introduction

Lignocellulosic biomass is often investigated as a biodegradable and renewable substitute for petroleum products because environmental problems such as pollution, global warming, and fuel resource tensions have arisen from the development of various industries [1,2].

Cellulose is the most plentiful polysaccharide and the basic substance in the cell walls of plants. It is derived from lignocellulosic biomass and is the main origin of organic compounds in nature [3]. This linear homopolymer which is composed of β-D glucopyranose (1–4 linkage) units with three free hydroxyl groups (C_2_, C_3_, C_6_), has great potential to be applied in various industries [4,5]. These -OH groups, as active agents and appropriate sites for chemical modifications, can be substituted with other groups such as amines, carboxyls, aldehydes, and phosphorus, to give cellulose outstanding properties. Many studies have elaborated on different cellulose sources and their various properties. There is a lengthy history of its use in both native and modified forms to achieve desired and particular properties [1,4,5,6,7,8,9,10,11,12,13]. For example, starch, chitosan, and particularly cellulose as bio-based materials are often used in flame-retardant products [3]. The density of cellulose and the elasticity of its crystalline sites can be reached up to 1.6 g cm^−3^ and 100–200 GPa, respectively. Furthermore, cellulose can be used to create arranged configurations through self-assembly, originating from its semi-crystalline nature [13]. Various forms of cellulose such as cellulose derivatives, regenerated cellulose, microcrystalline cellulose (MCC), cellulose nanocrystals (CNC), cellulose nanofibrils (CNF), and other modified forms of cellulose have been investigated by researchers both in academic laboratories and industrial settings [14,15].

The purpose of this review study is to summarize the widely used nanoscale materials totally derived from cellulose bio-polysaccharides, with an emphasis on their use as raw materials in the wood and paper industry. Moreover, as the waterproof and fireproof concepts of modified cellulosic and nanocellulosic substrates have only recently been noticed by researchers in the wood and paper industry, the necessity of using the abovementioned surfaces is investigated as coatings. Finally, the future challenge in this field, obtaining binary coatings on cellulosic substances, is mentioned.

## 2. Water Resistance and Fire Resistance Implications

Before addressing the main issue, it is necessary to comprehensively address water resistance and fire resistance concepts.

Following surface chemistry development, hydrophobic and superhydrophobic surfaces have presented potential applications such as anti-cohesion, self-cleaning, liquid separation, and printing and re-printing, which are fascinating from both academic and industrial viewpoints [16,17,18,19]. Before considering the abovementioned surfaces in detail, the wetting behavior is explained by three different modules as follows [20,21,22,23]:

(1) $$\mathrm{Young}\text{’}s\;\mathrm{equation}:\;\cos\;\theta =\frac{\gamma_{sv}-\gamma_{sl}}{\gamma_{lv}}$$

(2) $$\mathrm{Wenzel}\text{’}s\;\mathrm{equation}:\;r\left(\gamma_{sv}-\gamma_{sl}\right)=\gamma_{lv}\;\cos\;{\theta^{*}}_{w}\;$$

Cassie and Baxter’s equation: cos *θ* = *f*_1_ cos *θ*_1_+ *f*_2_ cos *θ*_2_ (3)

where γ*_Sv_*, γ*_Sl_*, and γ*_lv_* are the surface tension between solid-vapor, solid–liquid and liquid–vapor phases, respectively; *θ* and θ^*^_w_ are the contact angles exposed to a smooth and rough surface, respectively; *r* is defined as the roughness factor; *f*_1_, *f*_2_, *θ*_1_, and *θ*_2_ are, respectively, the surface area fraction and contact angle for substrates 1 and 2, which are valid for a non-homogeneous surface.

**Figure 1:**
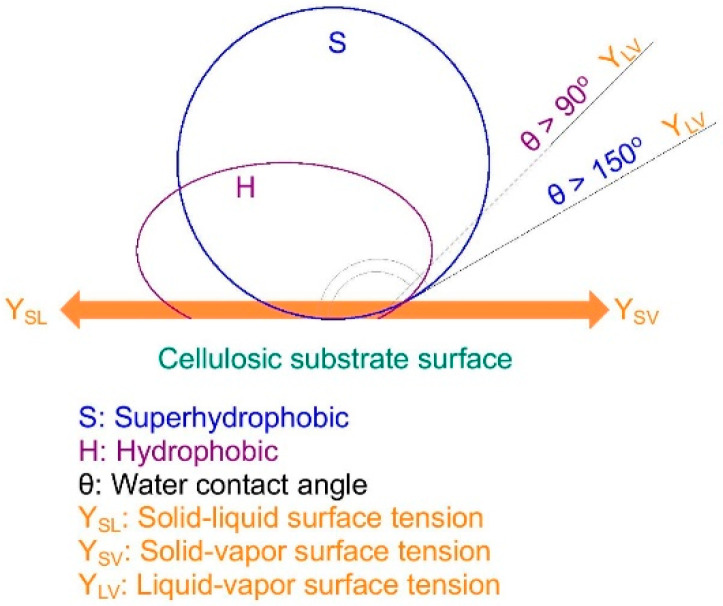
Illustration of hydrophobic and superhydrophobic surfaces based on water contact angle measurement.

The water contact angle (WCA), which is measured for liquid droplets, is the key to distinguishing hydrophobic surfaces from superhydrophobic ones. The sliding angle (SA), as another important parameter, is mostly used in conjunction with WCA for waterproof surfaces. More precisely, the higher WCA, the lower SA, and consequently, the better the water repellency. Sustainable and bio-inspired materials can be applied as hydrophobic and superhydrophobic agents, which provide sufficient durability versus liquid droplets. As shown in Figure 1, a hydrophobic surface presents a water contact angle of more than 90°, whereas a WCA of more than 150° represents a superhydrophobic surface [16,17,18,19].

The fully bio-inspired waterproof surfaces, which originate from biopolymers such as cellulose, starch, and proteins, have been demonstrated remarkable merits in terms of environmental concerns, biodegradability, sustainability, and reusability particularly than traditional synthetic polymers such as polylactic acid (PLA), polybutylene succinate (PBS), and polyethylene furanoate (PEF) for paper and packaging objectives. However, native forms of cellulosic derivatives, owing to their high water absorbability, require modification through coating or an impregnation process, surface treatments, plasticizing, etc. for water-resistant applications [2,4,5,6,7,8,9,11,12,17,18,19].

**Figure 2:**
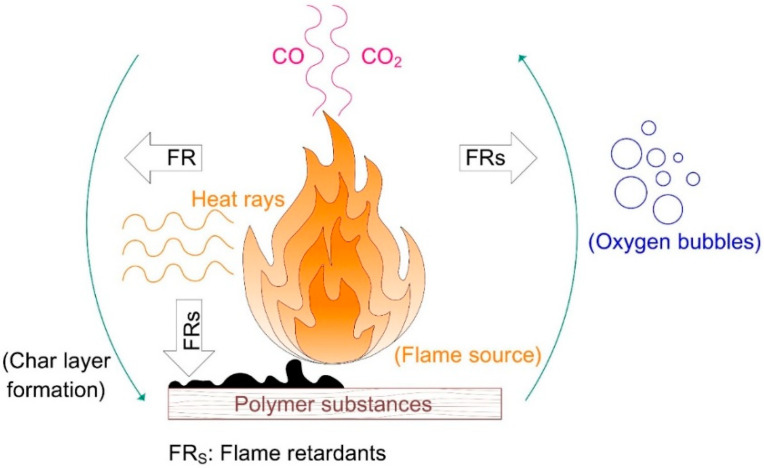
Functional mode of flame retardants.

Moreover, as observed in Figure 2, the determinant role of flame retardants is defined via the improvement of the self-stability of the polymer combustion cycle, which prevents ignition propagation either physically (cooling down, fuel dilution, protective layer formation) or chemically (gas phase, condensed phase). The flame extinguishing and ignition reduction are accomplished by the following procedures:

- Modifying the pyrolysis process, decreasing flammable volatiles, or increasing the formation of low-flammability gases, which act as a barrier layer between the polymer and sublayers.
- Isolating the flame and heat from oxygen in the air.
- Applying flame retardants or dilution agents.
- Declining the heat regression to the polymer, which hinders re-ignition by forming a protective barrier such as a char layer or intumescent coating when the polymers are subjected to heat sources.

Flame retardants are divided into two subcategories based on their operation mechanism, as follows:

Reactive: ignition inhibitors that are used during polymer synthesis (as monomers) or post-reaction processes.

Additives: combustion retardants, which are commonly applied during the polymer deformation process [3,23,24,25,26,27].

There are four common tests for evaluating combustion inhibitors:

▪ Limiting oxygen index (LOI)

The minimum content of oxygen in the air is measured by LOI. An LOI of less than 21% represents spontaneous ignition without further heating. The greater the limiting oxygen index, the better the flame-retardant properties.

▪ Vertical flammability (UL 94)

UL94 is a conventional method for fire propagation rate evaluation. The output data include the ignition duration, as well as flame spreading. Rates amongst V-0 to V-2 indicate combustion propagation. V-0 indicates that polymers are easily ignited and extinguished. V-1 and V-2 correspond to more time for flame spreading. No rate indicates no ignition.

▪ Cone calorimeter

Since the combustion process consumes oxygen, the oxygen concentration is recorded continuously during the test. The peak heat release rate is considered to be the major parameter for the assessment of fire hazards.

▪ Pyrolysis-combustion flow calorimeter (PCFC)

Data derived from this test include the total heat release, intensity, and temperature obtained from the peak heat release rate [3,10,24,26,27,28,29,30].

**Figure 3:**
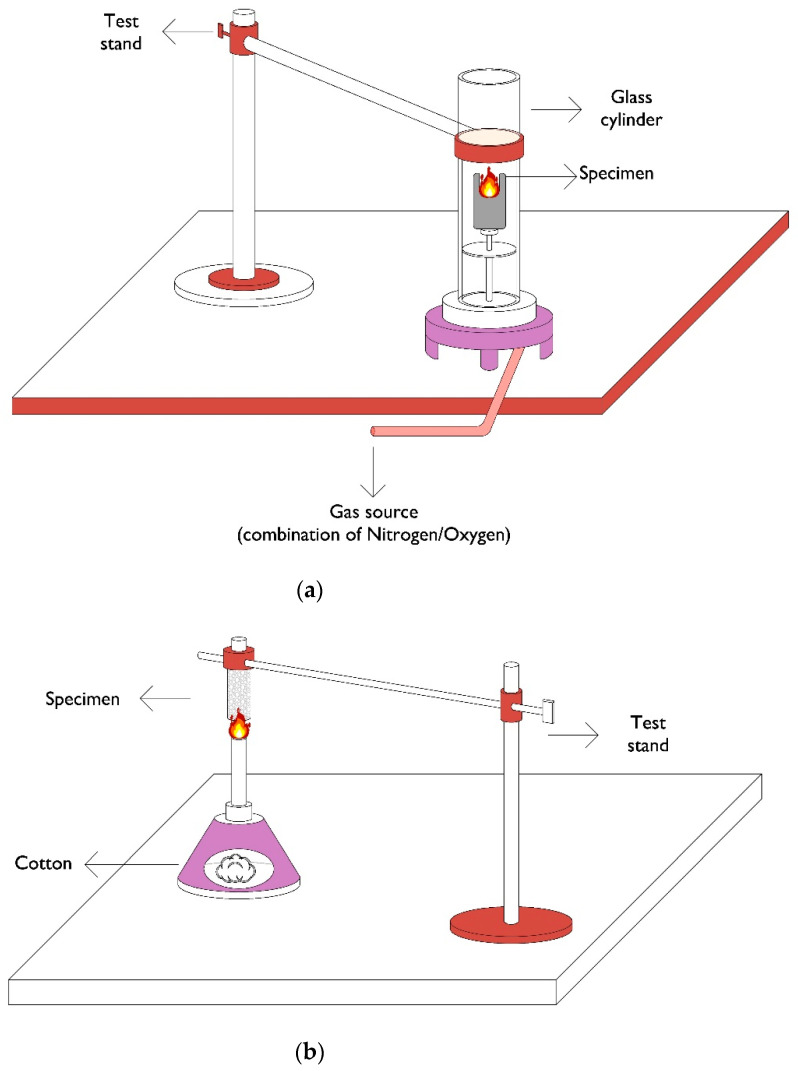

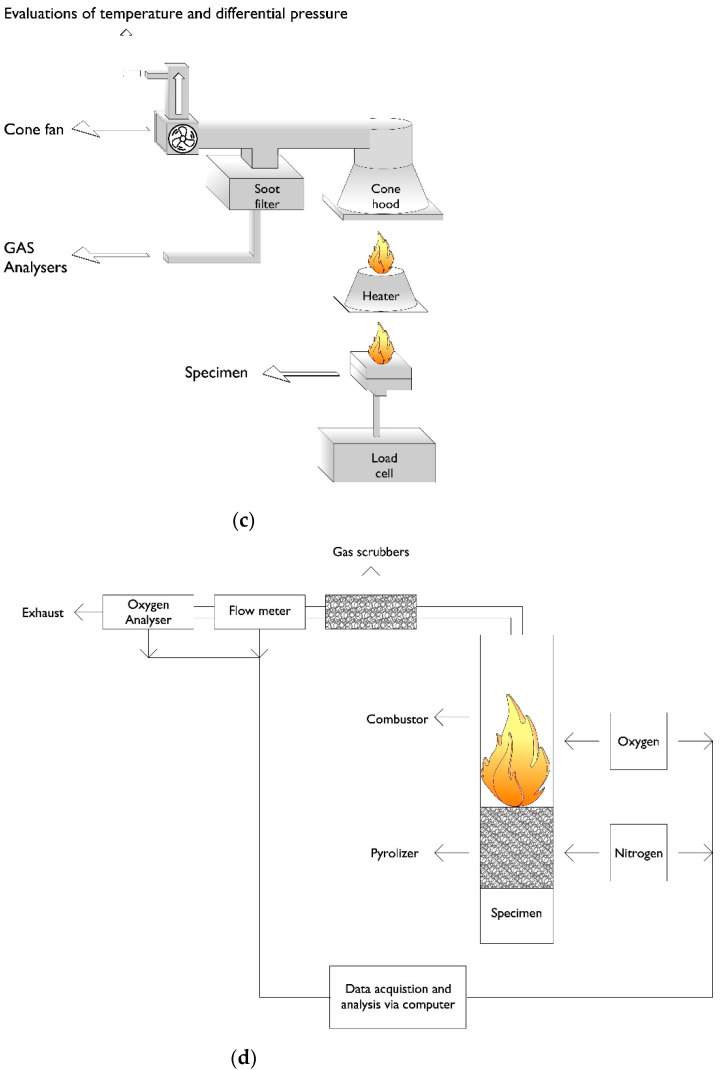
Different tests used to measure the effects of combustion inhibitors. Limiting oxygen index (**a**), vertical flammability (**b**), cone calorimeter (**c**), and pyrolysis-combustion flow calorimeter (**d**).

Some apparatuses that are commonly used for flame retardancy tests are shown graphically in Figure 3.

Recently, biobased fire inhibitors have grown exponentially due to the environmental limitations derived from plastic waste management. Even though engineering plastics such as polylactic acid (PLA), polybutylene terephthalate (PBT), poly trimethylene terephthalate (PTT), and polyethylene terephthalate (PET) have been commonly known as intrinsic fire retardant substances due to their self-extinguishing behavior, and appropriate dimensional and mechanical stability [3,23,24,25,26,27]. In this regard, four known biobased classifications, including carbohydrates, phenolic compounds, proteins, and lipids, have been introduced, which can be used in the naive or derivative form via biological or chemical modifications. Furthermore, functional groups for chemical modifications, suitable thermal stability, inherent fire resistance elements such as nitrogen, silicon, and phosphorus, polymer processing compatibility, and the ability to form the char barrier layer has been propounded as an efficiency indicator of biomolecules [3,10,24,26,27,28,29,30].

## 3. Prevalent Applicable Nanocellulosic Substances in the Wood and Paper Fields

Nanoscience refers to nanoscale materials (10^−9^ m), which exhibit at least one dimension with a size in the range of 1–100 nanometers. The Technical Association of Pulp and Paper Industries (TAPPI WI 3021) has proposed standard terms for nanocellulosic substances that are totally derived from nanocellulose. Nanocellulosic materials have been derived from lignocellulosic biomass and are classified into three major subcategories.

- Cellulose nanofibrils, introduced as CNF or NFC (*nanofibrillated cellulose*).
- Cellulose nanocrystals, known as CNC or NCC (nanocrystalline *cellulose*).
- Bacterial cellulose, displayed as BC or BNC (bacterial *nanocellulose*).

The abovementioned classification is mainly derived from their preparation procedure, function, dimensions, configuration, and cellulose origin [4,6,7,14,15].

Cellulose nanofibrils display long nanoscale structures due to interlaced and flexible nanofibrils, which usually have widths in the range of 4–20 nm and a length between 50–2000 nm. For the production of CNFs, which consist of crystalline and amorphous subregions, various mechanical and chemical techniques are applied in the presence or absence of pretreatment or posttreatment processes to convey the desired characteristics. The advantages of CNFs include appropriate processability, large specific surface, recyclability, biodegradability, low density, and eco-friendliness. They also have disadvantages such as high energy consumption during production, low drainage for papermaking, and poor compatibility with hydrophobic polymers, which arises from the hydrophilic nature of CNFs [14,15,31,32,33,34]. Due to the use of various modifications such as enzymatic pretreatment, acidic hydrolysis, carboxymethylation, and TEMPO (2,2,6,6-tetramethylpiperidine-1-oxyl radical) oxidation, the energy expenditures have been significantly reduced [1,4,7,8,15,31,32,33,34].

Lourenço et al. [8] considered the usage of CNFs using TEMPO oxidation and carboxymethylation individually as reinforcement fillers that promote the mechanical and optical features for printing and writing paper. Cheng et al. [10] investigated the fire retardancy of an aerogel containing CNFs and zinc borate through TEMPO oxidation. The addition of zinc borate to the admixture reduced the heat release rate and improved the thermal insulation. Yook et al. [7] reported that different kinds of CNFs modified using alkyl ketene dimer (AKD) and organosilanes have great potential to be applied as barrier coatings for packaging. A 10 g/m^2^ coating weight was the proper option that displayed great strength against water, air, and grease. Arbatan et al. [35] fabricated superhydrophobic paper that consisted of CNF/precipitated carbonate calcium (PCC) as a filler and AKD as a sizing agent and coated these layers on filter papers via dip-coating.

Cellulose nanocrystals produced by acidic hydrolysis exhibit lengths of 100–500 nm and diameters of 5–50 nm. The extraction process and cellulose origin strongly affect the CNC dimensions. Due to their higher crystallinity, CNCs are less flexible than CNFs [33,36,37]. There are problems related to using the CNCs in the papermaking industry despite their nano-dimensions. Accordingly, surface coatings have been proposed [14,38].

It was reported that due to water permeation amongst nanocrystals, CNC films are more prone to swelling than CNF films [14]. Some authors have proposed the use of film composites containing starch, a natural biopolymer, incorporated within CNCs. This composite was introduced as a promising alternative that could be utilized for eco-friendly, biodegradable, and cost-effective packaging [39,40,41]. Campano et al. [42] concluded that using CNC without pretreatment (only hydrolyzed), the process efficiency was increased twofold without aggravating the thermal characteristics, CNC dimension, or crystallinity. Peng et al. [43] highlighted that various drying procedures of CNCs and CNFs directly affected the thermal behavior and crystallinity of these nanomaterials. In this regard, a superior combination was ascribed to CNFs in terms of their thermal behavior and crystallinity using the spray-drying method.

Bacterial cellulose originates from the use of various kinds of bacteria through fermentation, which is affected by the culture medium containing sugar. The diameter of BC is in the range of 20–100 nm. Different types of enzymes have a decisive role as intermediates during BC manufacturing. Carbon and nitrogen have been introduced as two main feedstock in the culture medium for growing bacteria. In addition, BC is identified as one of the sources suitable for CNC fabrication. Some of the bacteria used for BC production include *Acetobacter xylinum* [44], *Gluconacetobacter xylinus* [45], *Komagataeibacter*, and *Zoogloea* [37,44,45,46].

It has been demonstrated that bacterial nanocellulose is non-toxic, especially when compared to CNF and CNC, which have some concerns surrounding their toxicity [47]. George et al. [44] declared that the thermal stability and mechanical properties were much greater for CNC derived from BC (*Acetobacter xylinum*) compared with CNC derived from acidic hydrolysis. Some researchers reported that BC remarkably reinforced the strength of paper-based substrates [37,48,49].

**Figure 4:**
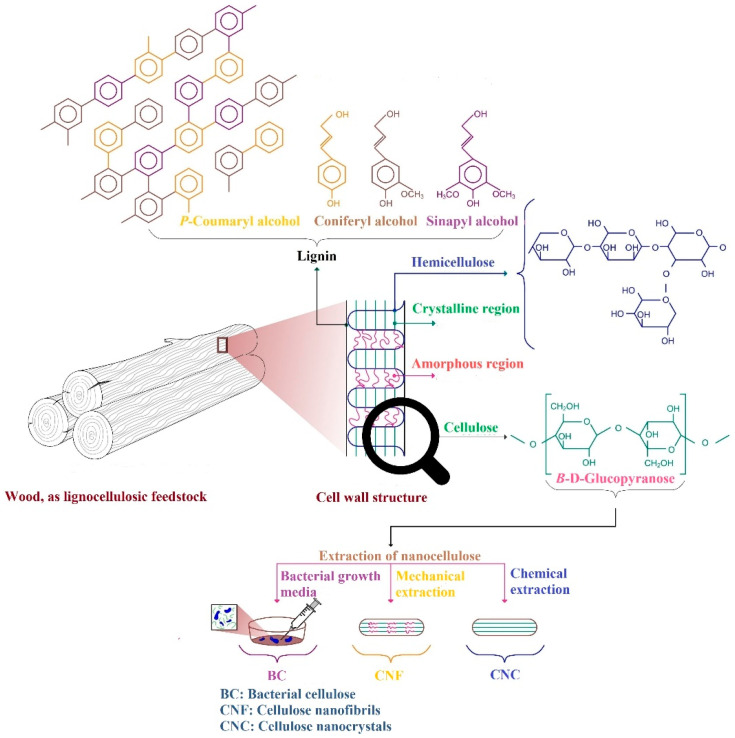
A schematic of cellulose biopolymer derivation from wood, which is converted to CNF, CNC, or BC, as the three major nanoscale forms of cellulose.

The production stages of the cellulose chains, CNF, CNC from wood, lignocellulosic biomass, and BC from bacterial cellulose, are illustrated in Figure 4.

Some of the most widely used methods of nanocellulose fabrication are summarized in Table 1.

## 4. Cellulose and Nanocellulose Substrates as Coating Layers

Sustainable and green cellulosic-based substances have become an indispensable part of daily life due to their widespread and distinguished roles in packaging, storage, and information transmission; however, due to the hydrophilic nature of cellulose, its structure can be easily destroyed by moisture and temperature. Therefore, bio-based, innovative, and green compounds have been appointed to improve the resistance against water droplets and fire, which are placed on cellulosic surfaces via different types of coating processes [70,71].

Paper and paperboards have been recognized as the most widely used substrates after plastics for packaging purposes. Cosmetics, pharmaceuticals, consumable goods, domestic appliances, and industrial and strategic commodities such as petrochemicals are relevant to the packaging industry. Paper-based products have raised attention due to their cost-effectiveness, accessibility, light-weight nature, proper mechanical features, and printing easiness for printing and packaging applications. Generally, grammage is determined as the basic factor that distinguishes paper from paperboard. According to this classification, papers present a basic weight of 7–150 g/m^2^, while a basic weight of more than 150 g/m^2^ is attributed to paperboard [72,73]. Pigments, binders, and fillers are introduced as the three principal components of coating layers on the surface of cellulose-based materials to endow them with barrier properties for various applications. Biopolymers such as polysaccharides, proteins, and lipids can be applied as the coating layer on paper and paperboard surfaces [58,72,73].

Different methods for the coating process are addressed as follows:

Extrusion, size-press, rod or bar, dip, and curtain coating.

Extrusion advantages include non-stop processing, the formation of a homogeneous coating layer, less cracking, and fewer fine pores for inorganic coatings, and solvent-free methods. Nevertheless, it suffers from large coating weight and polymer instability [73,74,75,76].

Extrusion is the only solvent-free coating procedure, and size-press, rod or bar, dip, and curtain coating are solvent-based coating methods on cellulosic surfaces. Size press is mostly applied for imposing liquid coatings onto solid substrates such as paper (less than 10% solid coat layer) on industrial scales. The limited solid content may lead to an insufficient surface coating. In contrast, a laboratory-scale rod coater can prepare coating layers with a uniform thickness. Dip coating, as a facile and rapid coating method, requires the precise control of the coat layer thickness and is used for liquid coating slurries on cellulosic underlayers. Recently, curtain coating has gained more attention in the industry due to the formation of a homogeneous coating layer that covers the surface entirely and provides a barrier against water vapor and gas [73,74,75,76,77].

Nanocellulose films have attractive properties such as biodegradability, renewability, high mechanical strength, and bio-based sources and have been applied as promising alternatives to fossil-based and synthetic polymers for cellulosic surface coatings [73,78]. Nanoscale materials can be used as barrier film reinforcements and as a component of paper-based coating formulas. Some authors have declared that nanocellulose coatings can provide a barrier that is resistant to water droplets, different gases such as carbon dioxide and oxygen, volatile components, and grease for food packaging [74,75,76,77,78,79,80,81].

## 5. The Necessity of Using Waterproof Surfaces for Cellulose-Based Substances

Cellulose-based substrates retain the hydrophilic nature of cellulose, exhibit a high permeability to gases, moisture, and grease than plastics. Therefore, to overcome these shortcomings, coatings or impregnation procedures have been used. Moreover, despite the high energy consumption and production costs, incorporation of nanocellulose derivatives to polymer matrix due to high resistance properties and transparency could be compensated the poor barrier effect of cellulose against moisture. Films containing nanofibrilated cellulose due to the fiber matrix stiffness exhibit more minor water uptake and prominent barrier features than cellulose microfibrillated films. In addition, CNC films, because of their more crystalline nature than CNF, form a great barrier layer against liquid droplets [6,7,14,15,31,32,33,34,35,36,37,67].

Low surface energy and surface roughness are two key factors to obtain a water-repellent surface [19,70,82,83]. Hydrophobicity is achieved by modifying the surface chemistry and wettability transition (liquid–solid interaction) on a cellulosic surface. In addition, water repellency can be obtained via surface modification by micro- and nanoparticles. Generally, a hydrophobic surface demonstrates low surface energy. Fluoro compounds are often applied for surface energy reduction, but their utilization is limited because they are not eco-friendly.

Adhesion and liquid penetration are decreased on waterproof surfaces, which leads to easy sliding of liquid droplets on solid surfaces. Self-cleaning capability is known as the most important function of water-repellent surfaces. Thus, liquid-dust adhesion is greater than liquid-solid surface adhesion, and most dust particles are eliminated by liquid droplets. The anti-fouling properties of water-resistant substrates are derived from their resistance to water and microorganism growth. Waterproof surfaces, due to their anti-adhesion and self-cleaning features, have great potential to be utilized for the preservation of ancient books and documents, as a coating layer in high-quality packaging, and pharmaceutical devices that are prone to infection, bacteria, and contaminated surfaces. Water-resistant papers have been investigated for security applications, especially currency notes, which are exposed to dust, microorganisms, and hand sweat, to increase the durability of banknotes against water and moisture [16,17,18,19,82,83]. Latthe et al. [84] investigated the use of hydrophobic silica nanoparticles via spray coating for various surfaces such as wood, building walls, fabrics, and banknotes. They obtained a WCA of 160° and a SA of 10°. The self-cleaning potential of the superhydrophobic surface was considerable. Ogihara et al. [85] prepared transparent and superhydrophobic paper through facile spray coating of nano-silica particles tailored with different kinds of alcohols. They obtained silica nano-components containing ethanol, in which the short hydrocarbon chain of ethanol demonstrated prominent water repellency. In another study, the same authors [18] demonstrated that paper coated with modified silica nanoparticles/alumina trihydrate/titanium dioxide followed by silane coupling agents exhibited significant waterproofing effects. Accordingly, papers deposited with modified nano-silica particles have great potential applications as water-repellent banknotes. Gao et al. [86] evaluated the waterproof properties of composite using two different silica nanoparticles with different diameters (7 and 14 nm), which were dispersed in polydimethylsiloxane. They observed a linear relationship between the concentration and water contact angle. Wang et al. [16] coated a cellulosic surface with nano-silica particles via a sol-gel process, followed by impregnation in hexadecyltrimethoxysilane solution to obtain non-toxic, cost-effective, and superhydrophobic paper (WCA > 150°).

## 6. The Necessity of Using Fireproof Surfaces for Cellulose-Based Substances

Paper and paper products, as the main feedstocks of lignocellulosic biomass, are prone to ignition when near flames and produce ash as the residual material. Thereupon, the development of fire resistance or flame retardancy is indispensable for their applications in various industries. Flame-retardant cellulosic substrates have fascinating potential applications in air transport, electronics, securities, and ammunition packaging in the military industry.

Fire inhibitors have different chemical structures, operation modes, and consumption levels. Halogenated flame retardants, which are operated through combustion prevention in the gas phase, were used for many years. Although they were highly efficient at low concentrations, their utilization has been limited due to smoke and toxic gas production in the last decade. Phosphorus-containing flame retardants operate as flame inhibitors through char layer formation, which reduces the fuel content and accumulates on the surface, where it acts as a barrier. The formation of an intumescent layer reduces the heating in the underlying substrate. Ammonium polyphosphate is an intumescent flame retardant. The range of 15–30 wt% is frequently considered for phosphorus-containing flame retardants. Nanoparticle-based flame retardants are rarely applied solely as ignition inhibitors, but if they are well-dispersed with other materials, they can be very effective at low amounts (1–5 wt%). An improved barrier effect is accompanied by a reduction in the peak of the heat release rate. Nanoclays, specifically montmorillonites and carbon nanotubes (CNTs), are recognized as the most commonly used nano-based flame retardants [3,10,24,25,26,27,28,29,30,87].

Recently, the polymer industry has turned to bio-inspired flame retardants derived from natural polymers such as cellulose, lignin, starch, or alginate, to reduce environmental impacts. The oxygen-rich structures of bio-polymers have propelled a relatively low release rate of heat during combustion and oftentimes form char barrier layers. The formation of a barrier layer on the cellulosic surfaces is achieved for all bio-inspired flame inhibitors, which improves the thermal behavior of polymers.

A char layer presents dual positive effects:

i. By maintaining a constant carbon atom content, which constitutes the polymer structure, and by reducing the volatiles and heat release rate during combustion.
ii. The case of CNF is demonstrated better thermal behavior compared with char layer acts as a protective barrier that decreases the heat transmission to the underlying polymers and modifies the flame diffusion kinetics via self-intumescent processes.

Onset thermal decomposition of CNF and CNC is reported 350 °C and 200–300 °C, respectively, which in the hemicellulose and lignin. CNC comprising low sulfate presents efficacy thermal stability. In addition, the incorporation of nanoscopic fillers in composite promotes flame retardancy, barrier characteristic, stiffness, and durability compared with fully polymeric materials [3,10,23,24,25,26,27,28,29,30].

Some studies have investigated the dual effects of biotechnology and nanoscience for introducing innovative flame retardants, e.g., the prominent efficiency of nanoparticles and phosphorus-based compounds on the thermal behavior of cellulose [3,10,29,30,69,88,89]. Ghanadpour et al. [90] found that the presence of phosphate groups in cellulose nanofibers (phosphorylated CNFs) when using diammonium phosphate and urea as flame retardants significantly improved the combustion inhibitor properties of CNFs.

## 7. Durable, Cost-Effective, and Dual-Function Hydrophobic/Fire-Inhibiting Coatings on Cellulosic Substances

Presently, the production of hydrophobic, flame retardant and eco-friendly cellulosic substances has become an immense challenge for various industries. For this reason, the advent of innovative compounds to simultaneously modify and strengthen against water and fire is essential, particularly for securities, high-quality packaging, and defense applications, which are directly subjected to combustion and liquid droplets. The development of flame retardants with tunable wettability is a fascinating theme that shows great potential for various applications [70,91,92,93,94]. Currently, the textile industry has taken significant steps to develop dual-function ignition inhibitors and waterproofs, but there is still a large gap for obtaining multifunctional features in the wood and paper industry.

A biobased cellulosic aerogel with improved strength with fire retardants by controlling its wettability was applied in a composite of reduced graphene oxide/carboxymethyl cellulose/borate as a cross-linking agent, have produced by Shahzadi et al. [94]. Si and Guo [70] produced a mixture of dopamine-silica trimethylsilyl modified by stearic acid and magnesium hydroxide and used it to develop superhydrophobic paper with improved flame retardancy. Sohbatzadeh et al. [95] introduced multifunctional, eco-friendly, and affordable coatings for firewood and achieved a water contact angle of more than 137.73° using polydimethylsiloxane and hexamethyldisiloxane. Moreover, some authors have mentioned the mixture of ammonium polyphosphate/methylmethacrylate/phosphorus nanoparticles/strontium doped with lanthanide and composite containing phosphonitrile functionalized by allyl, trimethylolpropane tris, and perfluoro-decyl trichlorosilane as multifunctional agents that can be applied as wood surface coatings [96,97,98].

As described above, although some studies have reported the dual effects of fire retardant and water repellency on wood surfaces, there is a need for much more accurate surveys of multifunctional effects using biobased polymers for high-consumption cellulosic surfaces such as paper and paperboard.

## 8. Conclusions and Outlook

Cellulosic-based substances, as the most commonly applied lignocellulosic biomass derivatives, play a fundamental and vital role in various industries. Nevertheless, cellulosic feedstocks suffer from severe structural changes when subjected to humidity and temperature, which greatly restrict their applications. Moreover, nanocellulosic derivatives present potentialities such as retention aids, strength, and stiffness accelerators in the processing and paper industry, composites, medicine, and various other fields.

However, due to the structural similarity of nanocellulosic substances and cellulose carbohydrates in terms of their wettability and thermal behavior, the modification and induction of flame retardancy and waterproof properties are important to tackle their shortcomings particularly for packaging, securities, and defense purposes. Thus, the advent of green and biobased materials opens novel gateways to obtain multifunctional effects. At present, significant advances have been made in the textile and wood industries in this field. More studies are required to develop multifunctional, cost-effective, and environmentally friendly procedures for paper and paperboard, as the two main applications of cellulosic materials. In this review, we have propounded the basic concepts such as water repellent surfaces, intumescent fire retardancy, and the potential of using cellulose and its nano-substrates for the medication of surfaces. Some innovative multifunctional processes have been addressed as future challenges for the application of lignocellulosic material surfaces, but more efforts are needed in this area.

## Figures and Tables

**Table 1:** Outstanding properties of commonly used procedures for nanocellulosic substance production.

Type of Method	Features	Advantages	Disadvantages	References
Grinding/Super grinding	The fibrillation process of fibers occurs through the passing of cellulose slurries between rotor and stator, which produce shear forces that separate the nanofibers	No need for additional mechanical pretreatment	Wood fibers can expunge the grooves of the discs Maintenance and replacement of discs are costly and time-consuming	[31,37,50,51]
High-pressure homogenization	This method involves suspension crossing via a narrow nozzle exposed to the high-pressure piston	Higher pressure gives a higher yield A homogeneous net is received Increasing the specific surface area	Homogenization is blocked by incomplete separation of nanofibers Irreversibility of changed fibers	[31,37,52,53,54,55]
Microfluidization	Microfluidizer operates with a constant shear force opposite to the homogenizer (constant pressure) Suspension is pumped with high pressure by a z-shaped channel	Nanofibers can be produced with a homogeneous size distribution	Process repetition (even up to 10 times) is required for better fibrillation	[15,31,37,56,57,58,59]
High-intensity ultrasonication	Known as the conventional mechanical lab-scale procedure in aqueous media in which the suspensions are subjected to hydrodynamic forces	The fibrillation process depends on the concentration of fiber, size of the fiber, time, and temperature	Low-scale production	[31,37,60,61,62,63,64]
Cryocrushing	Mechanical fibrillation occurs for frozen cellulose, which produces fibers with 0.1-1 μm diameters	The crushing process requires liquid nitrogen and low temperatures	High energy consumption Low efficiency Expensive	[34,37,50,65,66]
Steam explosion	Suspension is rapidly subjected to steam with intensifying pressure	Nanofibers are constituted by the swift release of pressure	Non-uniformity of the CNF quality	[34,37,67,68,69]

## Data Availability

The data presented in this study are available on request from the corresponding author.
